# Psychosocial predictors of depressive and anxiety symptoms among self-initiated older Chinese migrants: evidence from Chiang Mai, Thailand

**DOI:** 10.3389/fsoc.2026.1803605

**Published:** 2026-09-14

**Authors:** Xinyao Huang, Chawisa Suradom, Kelvin C. Y. Leung, Tinakon Wongpakaran, Rewadee Jenraumjit

**Affiliations:** 1 Master of Science Program (Mental Health), Multidisciplinary Interdisciplinary School, Chiang Mai University, Chiang Mai, Thailand; 2 Department of Psychiatry, Faculty of Medicine, Chiang Mai University, Chiang Mai, Thailand; 3 Specialty of Psychiatry, Faculty of Medicine and Health, University of Sydney, Sydney, NSW, Australia; 4 Department of Pharmaceutical Care, Faculty of Pharmacy, Chiang Mai University, Chiang Mai, Thailand

**Keywords:** acculturative stress, anxiety, depression, loneliness, older migrants, psychosocial factors, sense of mastery, social support

## Abstract

**Introduction:**

This cross-sectional study examined psychosocial factors associated with depressive and anxiety symptoms among self-initiated older Chinese migrants in Chiang Mai, Thailand.

**Methods:**

A total of 204 participants aged 60 years or older, who had migrated to Thailand at the age of 60 or later and had lived in Thailand for more than six months, completed structured questionnaires between December 2024 and February 2025. Measures included depressive and anxiety symptoms, acculturative stress, perceived social support, sense of mastery, and loneliness.

**Results:**

The prevalence of depressive symptoms was 29.9%, while 53.4% reported anxiety symptoms. Bivariate analyses indicated that greater acculturative stress and loneliness and lower perceived social support and mastery were associated with greater depressive and anxiety symptoms. In the fully adjusted regression models, greater acculturative stress, lower perceived social support, and lower mastery were associated with greater depressive symptoms, whereas greater acculturative stress and loneliness and lower perceived social support were associated with greater anxiety symptoms. Parsimonious hierarchical sensitivity analyses broadly supported these findings, although the unique association between loneliness and depressive symptoms was marginal, and the association between mastery and anxiety varied by model specification.

**Discussion:**

These findings highlight the importance of psychosocial factors in the mental health of older migrants. Interventions should address acculturative stress and loneliness while strengthening perceived control and social connectedness. Longitudinal research should examine how these factors and institutional conditions interact over time to shape mental health trajectories in later life.

## 1 Introduction

The global phenomenon of population aging represents not just a demographic shift, but a profound transformation that is reshaping social arrangements, intergenerational relationships, and migration patterns worldwide (United Nations, n.d.). In the twenty-first century, the increase in life expectancy, coupled with declining fertility rates, is resulting in unprecedented growth of older populations, particularly in Asia (Ogawa et al., 2021). These demographic changes pose complex challenges and opportunities, calling for sociological inquiry into how aging, migration, and health interact in rapidly evolving societies.

Traditionally, the sociology of aging has viewed later life as a time of accumulating life experiences, renegotiation of social roles, and balancing independence with increasing dependency (von Humboldt et al., 2024). However, for a growing number of individuals, later life is also encompassing migration, either as a pursuit of a better quality of life or as a response to economic or familial pressures. Unlike younger migrants, older adults migrate with deeply rooted identities and face unique challenges in adapting to new sociocultural environments, shaped by both structural and cultural factors in their countries of origin and destination (Baldassar et al., 2017). These dynamics have direct implications for the well-being, sense of belonging, and social citizenship of older migrants.

Mental health emerges as a critical concern for this demographic. It is increasingly recognized that mental health among older migrants is influenced not by individual disposition alone, but by broader social determinants such as social support, financial security, language proficiency, and experiences of discrimination. Recent meta-analyses report that depression is prevalent among older adults in China (about 20%), with even higher rates among vulnerable groups, such as “empty nesters” (Tang et al., 2021; Zhang et al., 2020). Among older Chinese migrants abroad, depressive and anxiety symptoms have also been reported as important mental health concerns. For example, studies among older Chinese adults in the Chicago area reported that 54.4% had depressive symptoms and 65.0% reported at least one anxiety symptom, respectively. These studies used different assessment approaches and criteria for depressive and anxiety symptoms (Dong et al., 2014a; Dong et al., 2014b). These findings suggest that aging and migration may jointly contribute to increased mental health risks among older migrants.

The concept of “double jeopardy” succinctly describes how older migrants confront both age-related vulnerabilities and the challenges of cultural adaptation in a new society (Bhatia et al., 2024; Ciobanu et al., 2017). Migration in late life often triggers shifts in identity and belonging, making older migrants experience a sense of “in-betweenness” in their homeland and host country (Klok et al., 2024). Social integration, mediated by factors such as social class, ethnicity, and gender, is further complicated by isolation, language barriers, unfamiliar institutional systems, and, at times, marginalization (King et al., 2017; Sasiwongsaroj and Husa, 2022). These realities can limit social participation, curtail agency, and increase psychological distress (Guo et al., 2019; Hendriks, 2015).

Research specifically addressing older migrants lived experiences and emotional well-being in East and Southeast Asian migration contexts remains comparatively limited (Au et al., 2024). As migration among older adults increases, it becomes increasingly important to understand how social stratification, transnational networks, acculturation processes, and local integration shape mental health (Bhatia et al., 2024).

Thailand presents a distinctive context for investigating these issues because it is an important destination for international retirement and lifestyle migration. UN-based estimates reported approximately 77,000 Chinese migrants residing in Thailand in 2020, with Chiang Mai identified as an important destination for lifestyle- and education-related migration (Siriphon and Banu, 2021). Existing research also indicates that older migrants’ experiences in Thailand are shaped by access to care, local institutions, and transnational living arrangements (He and Sasiwongsaroj, 2023; Sasiwongsaroj and Husa, 2022).

Grounded in the Stress Process Model (Avison et al., 2010; Pearlin et al., 1981) and Berry’s acculturation framework (Berry, 2006), this study examines how social and personal resources jointly influence mental health among older migrants. Key psychosocial factors—such as acculturative stress (Kosic, 2004), loneliness (Jang and Tang, 2022), sense of mastery (Gadalla, 2009), and perceived social support (Bilecen and Vacca, 2021)—are regarded not simply as individual traits but as emerging from social relationships, cultural capital, and the structural opportunities available to migrants in late life.

Accordingly, this research investigates the social, cultural, and psychosocial determinants of depressive and anxiety symptoms among older Chinese migrants in Thailand, focusing on the interplay of social, cultural, and psychological factors in shaping mental health outcomes. By centering on an underexplored demographic, this study seeks to inform both theoretical understanding and practical interventions to enhance the well-being of older migrant populations.

## 2 Materials and methods

### 2.1 Study design and participants

This cross-sectional study was conducted in Chiang Mai Province, Thailand, from December 2024 to February 2025. Participants were older Chinese migrants living in Thailand. Inclusion criteria were: (1) Older people who migrated to Thailand for long-term residence and had lived in Thailand for more than 6 months, (2) Migrated to Thailand at the age of 60 or older, (3) Ability to hear, read, write, and communicate sufficiently in Chinese. Exclusion criteria included incomplete responses or a history of diagnosed mental disorders, cerebrovascular disease, Parkinson’s disease, dementia, disability, being bedridden, or brain injury.

### 2.2 Sample size and sampling technique

The sample size was calculated using the formula for estimating a population proportion with specified precision (Lwanga and Lemeshow, 1991). The calculation assumed a reasonable estimated prevalence of mental health symptoms based on prior studies in similar populations, with a 95% confidence level and a 5% margin of error. The minimum required sample was 246. To allow for non-response, the target sample was increased to 270. A total of 204 complete responses were included in the final analysis, as shown in Figure 1. The final sample size was lower than both the minimum estimated requirement and the recruitment target because of recruitment constraints, which may have reduced statistical power and should be considered when interpreting the findings.

**Figure 1:**
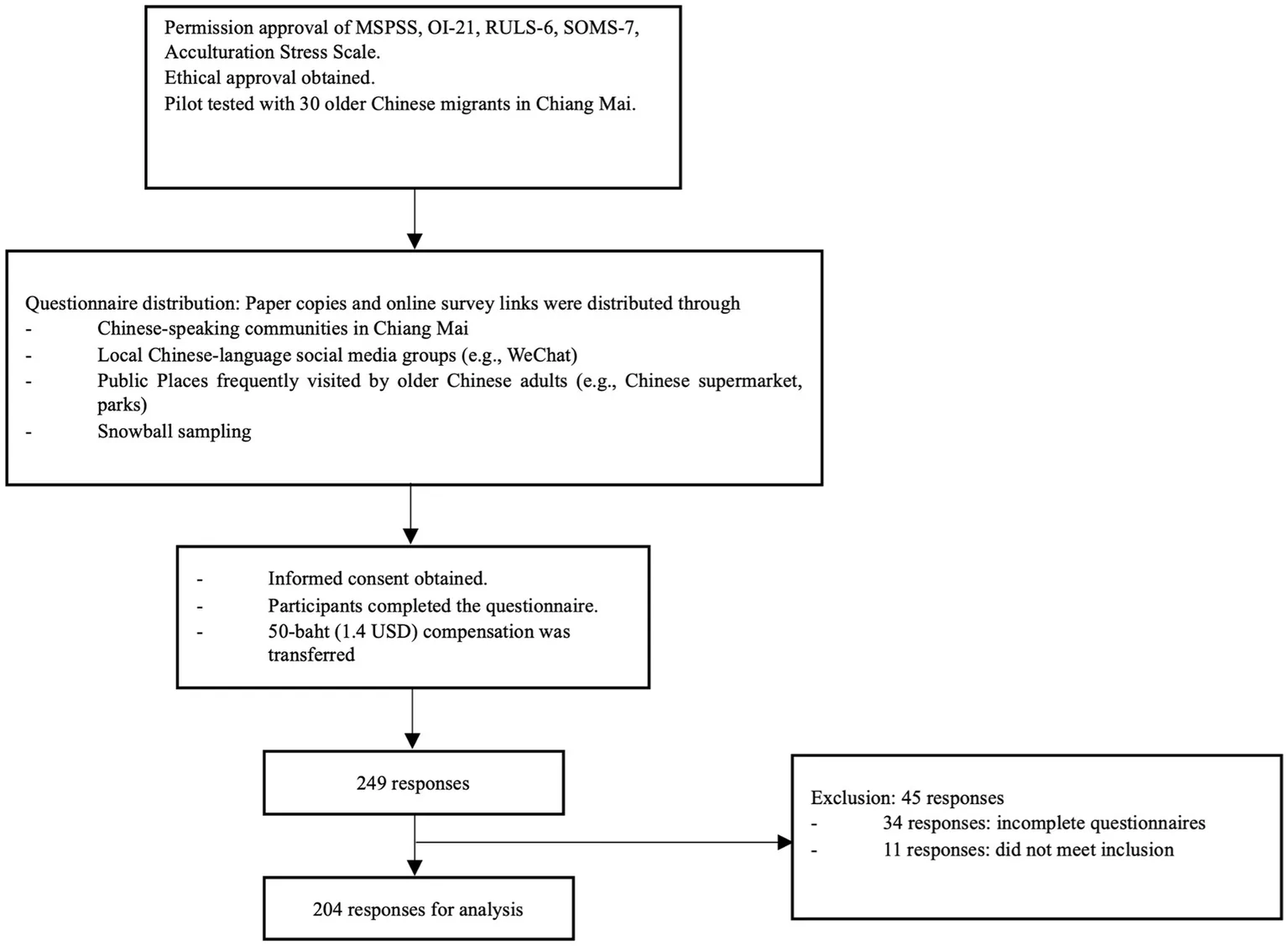
Flow chart of the study.

A convenience sampling method was employed due to the limited accessibility of the target population and the exploratory nature of the study.

### 2.3 Questionnaires and measurements

This study used a structured questionnaire with six sections to assess demographic characteristics, psychological well-being, and related psychosocial variables. Before the main data collection, a pilot test was conducted among older Chinese migrants in Chiang Mai to assess the clarity and applicability of the questionnaire. All scales used validated Chinese versions, and Cronbach’s alpha values for this sample are reported below.

#### 2.3.1 Sociodemographic data and information related to immigration

Data included age, gender, marital status, income, education, co-residence with children, number of household members, perceived health status, length of stay in Thailand, language proficiency (Chinese, Thai and English), and migration motivations.

#### 2.3.2 Perceived social support

Perceived social support was measured using the 12-item MSPSS, which assesses support from family, friends, and significant others on a 7-point Likert scale. The original scale demonstrated good internal consistency (Zimet et al., 1988). In this study, Cronbach’s alpha was 0.944.

#### 2.3.3 Loneliness

Loneliness was assessed using the 6-item RULS-6, with responses ranging from 1 (never) to 4 (always). Higher scores reflect greater loneliness. The scale showed good reliability in prior research (α = 0.83; Wongpakaran et al., 2020) and in this study (α = 0.878).

#### 2.3.4 Sense of mastery

The 7-item SOMS-7 assesses perceived control over one’s life using a 4-point scale from 1 (strongly disagree) to 4 (strongly agree), with higher scores reflecting stronger mastery. A previous Chinese-language study reported good internal consistency (α = 0.84; Yu and Zou, 2008). In this study, Cronbach’s alpha was 0.663, indicating relatively modest internal consistency.

#### 2.3.5 Acculturative stress

Acculturative stress was measured using the 10-item short version of the scale developed for the Chinese community in Kolkata, with responses on a 5-point scale from 1 (strongly disagree) to 5 (strongly agree). Higher scores indicate greater stress. Cronbach’s alpha was 0.87 (Biswas, 2022). In this study, Cronbach’s alpha was 0.903.

#### 2.3.6 Outcome Inventory-21

The Outcome Inventory-21 (OI-21, Chinese version) was used to assess psychological symptoms. This 21-item instrument includes subscales for depression, anxiety, somatization, and interpersonal difficulties. Each item is rated on a 5-point Likert scale (0 = not at all to 4 = almost always), with higher scores indicating more severe symptoms. In the present study, only the depression and anxiety subscales were used. Previous research reported good internal consistency for the depression and anxiety subscales of the OI-21 (Wongpakaran et al., 2022). In the present sample, Cronbach’s alpha coefficients were 0.858 for the depression subscale and 0.883 for the anxiety subscale. Scores of ≥8 on the respective subscales were used to indicate depressive symptoms and anxiety symptoms (Wongpakaran et al., 2022).

### 2.4 Ethical considerations

This study was approved by the Ethics Committee of the Faculty of Pharmacy, Chiang Mai University (Cert. No. 029/2024/E), valid from 5 November 2024 to 4 November 2025. All participants gave written informed consent and completed the questionnaire at their own pace.

### 2.5 Data analysis

All data were analyzed using SPSS version 26. Descriptive statistics (means, standard deviations, and frequencies) were used to summarize participants’ sociodemographic characteristics and psychological variables. Cronbach’s alpha coefficients were calculated to assess the internal consistency of each scale, with reverse scoring applied where necessary.

To examine differences in anxiety and depression, chi-square tests were used for categorical variables, and independent-sample t-tests were applied to compare mean scores across binary groups. All proposed variables were entered into a multiple linear regression model to identify predictors of depression and anxiety. A two-tailed *p*-value below 0.05 was considered statistically significant.

Additional sensitivity analyses were conducted using parsimonious hierarchical regression models to address concerns regarding model complexity relative to the sample size. Gender, age group, and perceived health status were entered in Step 1; length of residence in Chiang Mai was added in Step 2; and acculturative stress, perceived social support, sense of mastery, and loneliness were entered in Step 3. Changes in R^2^ were examined at each step, and variance inflation factors were used to assess multicollinearity.

## 3 Results

### 3.1 Participant characteristics

Most participants were aged 60–69 years (76.5%) and female (52.9%). The majority had a high level of education (73.0%) and were either married or in a partnered relationship (75.5%). Over half (58.8%) reported good or very good health, and 54.9% reported a low monthly income (Table 1).

**Table 1:** Sociodemographic characteristics of participants (*n* = 204).

Variable	*n* (%)
Age group	
60–69	156 (76.5)
≥70	48 (23.5)
Gender	
Female	108 (52.9)
Male	96 (47.1)
Education	
High	149 (73.0)
Low	55 (27.0)
Marital status	
Married / Partnered	154 (75.5)
Not married	50 (24.5)
Perceived health status	
Good/Very good	120 (58.8)
Average/Poor	84 (41.2)
Monthly income	
Moderate–high income	92 (45.1)
Low income	112 (54.9)

High education = high school, vocational school, bachelor’s degree or above; Low = middle school or below; Moderate–High income = monthly income ≥ 20,000 THB; Low income = < 20,000 THB; “Married/Partnered” includes those currently married or cohabiting.

### 3.2 Psychological measures

Anxiety and depression averaged 7.38 (SD = 4.88) and 5.01 (SD = 3.98), respectively. The mean scores were 60.72 for perceived social support, 43.61 for acculturative stress, 22.31 for sense of mastery, and 13.02 for loneliness (Table 2).

**Table 2:** Psychological measures (*n* = 204).

Variables	
Scores of psychological measures	Mean (SD)
Anxiety (range 0–24)	7.38 (4.88)
Depression (range 0–19)	5.01 (3.98)
Perceived social support (range 12–84)	60.72 (14.50)
Acculturative stress (range 16–76)	43.61 (12.00)
Sense of mastery (range 10–32)	22.31 (4.38)
Loneliness (range 6–24)	13.02 (4.30)

Values represent means and standard deviations (SD). Higher scores indicate higher levels of the respective construct.

### 3.3 Bivariate associations between key variables

As shown in Table 3, depressive symptoms were positively correlated with acculturative stress (r = 0.706, *p* < 0.001) and loneliness (r = 0.681, *p* < 0.001) and negatively correlated with perceived social support (r = −0.444, *p* < 0.001) and sense of mastery (r = −0.718, *p* < 0.001). Similarly, anxiety symptoms were positively correlated with acculturative stress (r = 0.686, *p* < 0.001) and loneliness (r = 0.722, *p* < 0.001) and negatively correlated with perceived social support (r = −0.401, *p* < 0.001) and sense of mastery (r = −0.647, *p* < 0.001).

**Table 3:** Correlation matrix between variables.

Variables	1	2	3	4	5	6	7	8	9	10	11	12	13
1. Age	—												
2. Co-residing family no.	−0.045	—											
3. Monthly income	0.055	0.032	—										
4. Chinese proficiency	−0.127	−0.136	−0.077	—									
5. Thai proficiency	0.028	0.292^**^	0.119	−0.087	—								
6. English proficiency	0.080	0.009	0.160^*^	−0.116	0.213^**^	—							
7. Length of residence	0.080	0.275^**^	0.129	0.023	0.473^**^	0.166^*^	—						
8. Social support	0.119	0.052	0.131	0.037	0.179^*^	0.162^*^	0.294^**^	—					
9. Acculturation stress	−0.002	−0.072	−0.392^**^	−0.026	−0.070	−0.112	−0.170^*^	−0.325^**^	—				
10. Sense of mastery	0.075	0.016	0.268^**^	0.037	0.024	0.127	0.051	0.354^**^	−0.689^**^	—			
11. Loneliness	0.073	−0.138^*^	−0.273^*^	−0.049	−0.179^*^	−0.152^*^	−0.170^*^	−0.389^**^	0.735^**^	−0.690^**^	—		
12. Anxiety	0.016	0.035	−0.273^**^	−0.124	−0.058	−0.104	−0.034	−0.401^**^	0.686^**^	−0.647^**^	0.722^**^	—	
13. Depression	0.048	−0.074	−0.278^**^	−0.141^*^	−0.121	−0.127	−0.135	−0.444^**^	0.706^**^	−0.718^**^	0.681^**^	0.834^**^	—

*p < 0.05, **p < 0.01, ***p < 0.001.

### 3.4 Associations between predictors and mental health symptoms

As shown in Table 4, higher acculturative stress (B = 0.091, β = 0.276, *p* < 0.001) was associated with greater depressive symptoms, whereas greater perceived social support (B = −0.039, β = −0.140, *p* = 0.006), greater sense of mastery (B = −0.285, β = −0.313, *p* < 0.001), higher Chinese proficiency (B = −6.193, β = −0.109, *p* = 0.020), and health insurance coverage (B = −0.452, β = −0.105, *p* = 0.036) were associated with fewer depressive symptoms. Loneliness was not independently associated with depressive symptoms in the fully adjusted model (B = 0.133, β = 0.144, *p* = 0.072).

**Table 4:** Multiple regression models examining factors associated with depressive and anxiety symptoms.

	Depression				Anxiety			
Predictor	B	SE	β	*p*-value	B	SE	β	*p*-value
(Constant)	15.640	3.851		<0.001	5.130	4.942		0.301
Gender	0.120	0.353	0.015	0.735	0.534	0.453	0.055	0.240
Age	0.448	0.440	0.048	0.311	−0.196	0.565	−0.017	0.729
District of residence	−0.005	0.034	−0.006	0.889	0.006	0.043	0.007	0.883
Marital status	−0.809	0.453	−0.088	0.076	−0.436	0.582	−0.039	0.454
Education level	−0.394	0.418	−0.044	0.347	−0.791	0.536	−0.072	0.142
Living with children	−0.269	0.482	−0.034	0.577	0.839	0.618	0.086	0.177
Co-residing family no.	0.013	0.291	0.003	0.964	0.798	0.374	0.130	0.034
Perceived health status	−0.727	0.403	−0.090	0.073	−0.642	0.518	−0.065	0.217
Chinese proficiency	−6.193	2.645	−0.109	0.020	−7.161	3.394	−0.103	0.036
Thai proficiency	−0.282	0.448	−0.035	0.530	−0.398	0.575	−0.040	0.491
English proficiency	0.039	0.421	0.004	0.927	0.275	0.541	0.025	0.611
Monthly income	0.268	0.399	0.034	0.503	−0.009	0.513	−0.001	0.986
Health insurance	−0.452	0.213	−0.105	0.036	−0.447	0.274	−0.085	0.104
Length of residence	0.374	0.221	0.091	0.092	0.762	0.283	0.151	0.008
Visa type	−0.413	0.523	−0.038	0.431	0.633	0.671	0.048	0.347
Perceived social support	−0.039	0.014	−0.140	0.006	−0.039	0.018	−0.117	0.029
Acculturative stress	0.091	0.024	0.276	<0.001	0.115	0.031	0.284	<0.001
Sense of mastery	−0.285	0.066	−0.313	<0.001	−0.138	0.085	−0.123	0.105
Loneliness	0.133	0.074	0.144	0.072	0.438	0.095	0.386	<0.001
R^2^	0.699				0.669			
F	13.906 (29,174)				12.139 (29,174)			

B = unstandardized regression coefficient; β = standardized regression coefficient; *p* = significance level.

For anxiety symptoms, higher acculturative stress (B = 0.115, β = 0.284, *p* < 0.001), greater loneliness (B = 0.438, β = 0.386, *p* < 0.001), a greater number of co-residing family members (B = 0.798, β = 0.130, *p* = 0.034), and longer residence in Chiang Mai (B = 0.762, β = 0.151, *p* = 0.008) were associated with greater anxiety symptoms. Greater perceived social support (B = −0.039, β = −0.117, *p* = 0.029) and higher Chinese proficiency (B = −7.161, β = −0.103, *p* = 0.036) were associated with fewer anxiety symptoms. Sense of mastery was not independently associated with anxiety symptoms in the fully adjusted model (B = −0.138, β = −0.123, *p* = 0.105).

Additional parsimonious hierarchical regression analyses were conducted as sensitivity analyses (Table 5). For depressive symptoms, Step 1 explained 7.6% of the variance (R^2^ = 0.076, adjusted R^2^ = 0.062, *p* = 0.001). Adding length of residence in Step 2 did not significantly improve the model (ΔR^2^ = 0.014, *p* = 0.083). The psychosocial variables added in Step 3 explained an additional 55.9% of the variance (ΔR^2^ = 0.559, *p* < 0.001). The final model explained 64.8% of the variance in depressive symptoms (adjusted R^2^ = 0.634). Higher acculturative stress (B = 0.099, β = 0.298, *p* < 0.001) and older age group (B = 0.829, β = 0.089, *p* = 0.047) were associated with greater depressive symptoms, whereas higher perceived social support (B = −0.047, β = −0.173, *p* < 0.001) and greater sense of mastery (B = −0.323, β = −0.355, *p* < 0.001) were associated with fewer depressive symptoms. Loneliness showed a marginal unique association with depressive symptoms (B = 0.125, β = 0.135, *p* = 0.056).

**Table 5:** Hierarchical regression sensitivity analyses for depressive and anxiety symptoms.

Panel A. Hierarchical model summary							
Outcome	Model	Variables added	R^2^	Adjusted R^2^	ΔR^2^	F change (df1, df2)	*p*
Depression	1	Gender, age, perceived health	0.076	0.062	0.076	5.467 (3, 200)	0.001
	2	Length of residence	0.090	0.071	0.014	3.034 (1, 199)	0.083
	3	Four psychosocial factors	0.648	0.634	0.559	77.469 (4, 195)	<0.001
Anxiety	1	Gender, age, perceived health	0.085	0.071	0.085	6.165 (3, 200)	<0.001
	2	Length of residence	0.085	0.066	<0.001	0.044 (1, 199)	0.834
	3	Four psychosocial factors	0.623	0.608	0.539	69.726 (4, 195)	<0.001

Panel B. Final eight-predictor models								
Predictor	Depression B	SE	β	*p*	Anxiety B	SE	β	*p*
Constant	8.012	2.400		0.001	0.791	3.042		0.795
Gender	0.185	0.345	0.023	0.592	0.433	0.437	0.044	0.323
Age group	0.829	0.414	0.089	0.047	0.141	0.525	0.012	0.789
Perceived health status	−0.324	0.362	−0.040	0.371	−0.742	0.459	−0.075	0.107
Length of residence	0.009	0.187	0.002	0.961	0.642	0.237	0.127	0.007
Acculturation stress	0.099	0.023	0.298	<0.001	0.113	0.029	0.279	<0.001
Perceived social support	−0.047	0.013	−0.173	<0.001	−0.048	0.017	−0.142	0.006
Sense of mastery	−0.323	0.060	−0.355	<0.001	−0.154	0.075	−0.138	0.043
Loneliness	0.125	0.065	0.135	0.056	0.418	0.082	0.369	<0.001

Model 1 included gender, age group, and perceived health status. Model 2 additionally included length of residence. Model 3 additionally included acculturative stress, perceived social support, sense of mastery, and loneliness. B = unstandardized regression coefficient; SE = standard error; β = standardized regression coefficient; ΔR^2^ = change in explained variance. VIF values for the predictors in the final models ranged from 1.04 to 2.73, indicating no severe multicollinearity.

For anxiety symptoms, Step 1 explained 8.5% of the variance (R^2^ = 0.085, adjusted R^2^ = 0.071, *p* < 0.001). Adding length of residence in Step 2 did not significantly increase the explained variance (ΔR^2^ < 0.001, *p* = 0.834). The psychosocial variables entered in Step 3 explained an additional 53.9% of the variance (ΔR^2^ = 0.539, *p* < 0.001). The final model explained 62.3% of the variance in anxiety symptoms (adjusted R^2^ = 0.608). Longer residence (B = 0.642, β = 0.127, *p* = 0.007), higher acculturative stress (B = 0.113, β = 0.279, *p* < 0.001), and greater loneliness (B = 0.418, β = 0.369, *p* < 0.001) were associated with greater anxiety symptoms. Higher perceived social support (B = −0.048, β = −0.142, *p* = 0.006) and greater sense of mastery (B = −0.154, β = −0.138, *p* = 0.043) were associated with fewer anxiety symptoms. VIF values for the predictors in the final models ranged from 1.04 to 2.73, indicating no severe multicollinearity.

## 4 Discussion

This study examines depressive and anxiety symptoms among self-initiated older Chinese migrants in Southeast Asia, demonstrating that psychological distress remains prevalent even among individuals who migrate voluntarily and report relatively stable living conditions. The findings challenge simplified assumptions that late-life migration is inherently protective for mental health and point to the complex interaction of psychological, cultural, and contextual factors shaping emotional well-being in later life.

In the present sample, 29.9% reported depressive symptoms and 53.4% reported anxiety symptoms. Comparisons with previous studies should be made cautiously because estimates vary across populations, instruments, and thresholds. For example, a study of community-dwelling older adults in Hunan, China, reported a higher prevalence of depressive symptoms but a lower prevalence of anxiety symptoms than observed in the present study (Lu et al., 2023). Similarly, anxiety symptoms were more prevalent in the present sample than among Vietnamese migrants in Japan, whereas depressive symptoms were slightly less prevalent (Yamashita et al., 2023). These differences highlight the importance of population characteristics and measurement approaches when comparing symptom prevalence across studies.

Psychological factors emerged as central correlates of mental health outcomes. Acculturative stress was a strong and consistent predictor of both depressive and anxiety symptoms, aligning with Berry’s acculturation framework, which emphasizes the emotional costs of sustained cultural negotiation in later life (Berry, 2006). This finding underscores that cultural adaptation challenges may persist well beyond the initial migration period and can constitute a chronic source of psychological strain among older migrants.

Perceived social support showed a consistent inverse association with both depressive and anxiety symptoms across the full and parsimonious models, consistent with evidence that interpersonal resources are relevant to migrant health (Bilecen and Vacca, 2021). Loneliness showed a strong and consistent association with anxiety. Its unique association with depressive symptoms was not statistically significant in either the full model (*p* = 0.072) or the parsimonious sensitivity model (*p* = 0.056), although the latter approached the conventional significance threshold. This pattern does not indicate an absence of a bivariate relationship between loneliness and depressive symptoms. Rather, it suggests that loneliness shares substantial explanatory variance with acculturative stress, perceived social support, and mastery. This interpretation is broadly consistent with previous evidence linking loneliness with depressive and anxiety symptoms among middle-aged and older populations (Dong et al., 2025).

Sense of mastery was consistently associated with lower depressive symptoms in both the full and parsimonious models. Its association with anxiety varied by model specification: it was not statistically significant in the full model but reached significance in the parsimonious sensitivity model. This difference may reflect changes in the unique variance attributed to mastery after less theoretically central covariates were removed. Overall, the findings suggest that mastery may represent an important psychological resource for mental health in later life, although its unique association with anxiety should be interpreted cautiously (Bian et al., 2024; Shin and Park, 2024).

Differences in statistical significance across the full and parsimonious models likely reflect changes in the unique variance attributed to each predictor after adjustment for conceptually overlapping psychosocial constructs. Regression coefficients represent the unique adjusted association of each variable rather than its simple bivariate relationship with the outcome. Although these constructs were correlated, the collinearity diagnostics from the parsimonious models did not indicate severe multicollinearity.

Several contextual and migration-related factors were specifically associated with anxiety. A greater number of co-residing family members was linked to higher anxiety levels, suggesting that multigenerational living arrangements may introduce relational demands or role expectations that elevate emotional tension in later life (Hanum et al., 2024). In addition, longer duration of residence was associated with increased anxiety, indicating that extended time in the host country does not necessarily translate into emotional adjustment or reduced stress (Honkaniemi et al., 2020). These findings challenge linear assumptions about adaptation over time and suggest that cumulative stressors or unmet expectations may intensify anxiety rather than alleviate it.

Language-related factors also warrant consideration. Lower Chinese proficiency was associated with both depressive and anxiety symptoms in the fully adjusted models, whereas Thai proficiency was not independently associated with either outcome. Because Chinese proficiency was measured using a broad self-reported indicator, the mechanism underlying this unexpected association remains unclear. It may reflect literacy, dialect-related communication, social participation, or other unmeasured characteristics and should be examined in future research rather than interpreted as evidence of host-country language barriers.

Finally, institutional conditions remain relevant to understanding psychological distress among older migrants. In the fully adjusted model, health insurance coverage showed a small inverse association with depressive symptoms (β = −0.105, *p* = 0.036), although this variable was not included in the parsimonious sensitivity model. Thailand has different health-coverage pathways for migrant populations, including employment-linked social security, migrant health insurance arrangements, and private coverage (World Health Organization Thailand, n.d.). However, the present study assessed only whether participants had health insurance and did not distinguish among public, employment-based, private, or overseas insurance schemes. The observed association should therefore be interpreted cautiously and does not establish which type of coverage may be related to mental health.

### 4.1 Implications

The findings underscore the importance of addressing psychosocial needs alongside healthcare access. Culturally and linguistically appropriate services may help older migrants manage acculturative stress, maintain supportive relationships, and reduce social isolation. Because the study did not distinguish among insurance schemes or directly assess healthcare utilization, future interventions and research should consider insurance type, continuity of coverage, accessibility of services, and out-of-pocket costs rather than treating insurance status as a uniform measure of institutional inclusion.

### 4.2 Future directions

Future research may further examine how institutional access, language-related resources, and psychosocial factors interact over time to influence mental health among older migrants. Longitudinal and mixed-methods approaches could help clarify how acculturative stress, social participation, and perceived autonomy change with increasing length of residence. Comparative studies across migrant groups and destination contexts may also provide insight into whether the patterns observed in Thailand reflect broader processes of later-life migration.

### 4.3 Limitations

Several limitations should be noted. The cross-sectional design restricts causal interpretation of the observed associations. The convenience sample was drawn from a specific group of older Chinese migrants residing in Chiang Mai, which may limit generalizability to other migrant populations or settings. The final sample of 204 was lower than the minimum estimated requirement of 246 and the recruitment target of 270, which may have reduced statistical power, particularly for detecting small unique effects of individual predictors. However, the overall regression models demonstrated substantial explanatory power (R^2^ = 0.699 for depressive symptoms and R^2^ = 0.669 for anxiety symptoms). In addition, the internal consistency of the sense of mastery scale was relatively modest in this sample (Cronbach’s α = 0.663), which may have reduced measurement precision and could attenuate or destabilize estimated associations involving mastery; findings related to mastery should therefore be interpreted cautiously. Health insurance was measured as a general coverage indicator without distinguishing public, employment-based, private, or overseas insurance schemes, limiting interpretation of its association with depressive symptoms.

The psychosocial predictors were moderately to strongly correlated, reflecting conceptual overlap among acculturative stress, social support, mastery, and loneliness. Although VIF values in the parsimonious models did not indicate severe multicollinearity, shared variance may have influenced the magnitude and statistical significance of their unique regression coefficients. Findings that varied across model specifications, particularly the associations of loneliness with depressive symptoms and mastery with anxiety symptoms, should therefore be interpreted cautiously.

## 5 Conclusion

Depressive and anxiety symptoms among older Chinese migrants in Thailand were associated with a combination of psychosocial and contextual factors. Acculturative stress and perceived social support showed relatively consistent associations across the full and parsimonious models, whereas the associations of loneliness and mastery varied by outcome and model specification. These findings support the importance of culturally appropriate mental health services that address adaptation-related stress and social resources.

## Data Availability

The raw data supporting the conclusions of this article will be made available by the authors, without undue reservation.
